# PACE – the first placebo controlled trial of paracetamol for acute low back pain: statistical analysis plan

**DOI:** 10.1186/1745-6215-14-248

**Published:** 2013-08-09

**Authors:** Christopher M Williams, Chris G Maher, Jane Latimer, Andrew J McLachlan, Mark J Hancock, Richard O Day, Laurent Billot, Chung-Wei Christine Lin

**Affiliations:** 1 The George Institute for Global Health and Sydney Medical School, University of Sydney, PO Box M201, Missenden Rd, 2040 Camperdown, NSW, Australia; 2 Faculty of Pharmacy and Centre for Education and Research in Ageing, University of Sydney, 2006 Sydney, NSW, Australia; 3 Faculty of Human Sciences, Macquarie University, 75 Talavera Rd, 2113 Sydney, NSW, Australia; 4 Clinical Pharmacology UNSW and St Vincent’s Hospital, 2010 Darlinghurst, NSW, Australia

**Keywords:** Acetaminophen, Back pain, Paracetamol, Statistical analysis plan, Randomised controlled trial

## Abstract

**Background:**

Paracetamol (acetaminophen) is recommended in most clinical practice guidelines as the first choice of treatment for low back pain, however there is limited evidence to support this recommendation. The PACE trial is the first placebo controlled trial of paracetamol for acute low back pain. This article describes the statistical analysis plan.

**Results:**

PACE is a randomized double dummy placebo controlled trial that investigates and compares the effect of paracetamol taken in two regimens for the treatment of low back pain. The protocol has been published. The analysis plan was completed blind to study group and finalized prior to initiation of analyses. All data collected as part of the trial were reviewed, without stratification by group, and classified by baseline characteristics, process of care and trial outcomes. Trial outcomes were classified as primary and secondary outcomes. Appropriate descriptive statistics and statistical testing of between-group differences, where relevant, have been planned and described.

**Conclusions:**

A standard analysis plan was developed for the results of the PACE study. This plan comprehensively describes the data captured and pre-determined statistical tests of relevant outcome measures. The plan demonstrates transparent and verifiable use of the data collected. This *a priori* plan will be followed to ensure rigorous standards of data analysis are strictly adhered to.

**Trial registration:**

Australia and New Zealand Clinical Trials Registry ACTRN12609000966291

## Background

The PACE study is the largest clinical trial to date of paracetamol treatment for patients with low back pain. Here, we describe the study’s pre-determined statistical analysis plan, which was finalized prior to analyzing the data set, and to which the investigators will adhere in analyzing the data from the trial.

The statistical analysis plan was completed and signed as approved by the study investigators on 3 June 2013. Participant recruitment was completed in November 2012, and final participant follow-up was completed in March 2013. Following data integrity checks the database will be locked (June 2013) and the statistical analysis specified in the statistical analysis plan will be performed in June 2013.

### Study overview

#### Design

The PACE study is a randomized double dummy placebo controlled trial that investigates and compares the effect of paracetamol taken in two regimens for the treatment of low back pain. The study is prospectively registered (ACTRN12609000966291) and a full protocol has been published [1]. The study enrolled 1,650 people seeking care for acute low back pain. All participants received advice to stay active and reassurance of a favorable prognosis. In addition, participants were randomized to receive study medication containing either active paracetamol or placebo. Participants were asked to take the study medication until the primary endpoint ‘recovery from back pain’ or for a maximum of four weeks after randomization. Follow-up lasted for three months.

#### *Inclusion criteria*

Patients were eligible for inclusion in the study if all of the following criteria were met:

• Had a primary complaint of pain in the area between the 12th rib and buttock crease, with or without leg pain;

• Experienced a new episode of low back pain, preceded by a period of at least one month without low back pain;

• Had low back pain of less than six weeks’ duration (in accordance with the Cochrane Collaboration Back Review Group definition for ‘acute’ pain);

• Had low back pain severe enough to cause moderate pain (as measured by adaptations of item 7 of the Short Form-36 Health Survey;

• Did not have known or suspected serious spinal pathology (for example, metastatic, inflammatory or infective diseases of the spine, cauda equina syndrome, spinal fracture);

• Were not currently taking recommended regular doses of analgesics, including paracetamol;

• Had not undergone spinal surgery within the preceding six months;

• Did not report serious co-morbidities preventing prescription of paracetamol, for example, liver or renal failure;

• Were not currently taking psychotropic medication for a health condition considered to prevent reliable recording of study information;

• Were not pregnant or planning to become pregnant during the treatment period.

#### *Objectives*

The primary aim of the PACE study is to establish if taking paracetamol results in more rapid recovery from acute low back pain than placebo.

The specific hypotheses to be tested are that:

1. Participants taking time-contingent paracetamol (4 g per day in divided doses) will have a more rapid recovery than those receiving placebo paracetamol.

2. Participants taking ‘as required’ paracetamol (up to 4 g per day) will have a more rapid recovery than those receiving placebo paracetamol.

3. Participants taking time-contingent paracetamol will have a more rapid recovery than those taking ‘as required’ paracetamol.

Secondary aims of the study are to establish if taking paracetamol in a time-contingent manner or ‘as required’ is more effective in reducing pain and disability and improving function, global rating of change, sleep quality and quality of life than placebo.

A separate analysis plan will be detailed for a health economic analysis and is not included in this manuscript.

#### *Unblinding*

The statistical analysis plan was written blinded to treatment allocation. Treatment allocations are stored in a secured location. Statistician(s) will remain blinded and work with a dummy coded variable for treatment allocation until the statistical computer code has been validated.

### Definition of outcome variables

#### *Definition of primary outcome*

The primary outcome is the number of days until recovery from low back pain. Self-reported pain scores based on a pain rating scale (0 to 10, where 0 is ‘no pain’ and 10 is the ‘worst possible pain’) were recorded for up to 12 weeks in a daily pain diary to calculate days to recovery. Participants were asked to return the diary after they had recovered or 12 weeks had elapsed.

Recovery is defined in two possible ways. The first is sustained recovery: the first day with a pain score of 0 or 1 on a 0 to 10 pain rating scale that is maintained for seven consecutive days. The second is first recovery: the first day that the participant has a pain score of 0 or 1 on a 0 to 10 pain scale.

For the purpose of the primary analysis, the first definition will used. This definition was selected based on consensus among study investigators that seven days pain-free is more meaningful to patients than just one day. The second definition will be used in a sensitivity analysis to confirm the robustness of the primary analysis.

#### *Definition of secondary outcome*

The secondary low back pain outcomes to be considered are:

• Pain in last 24 hours: measured using a numerical rating (0 to 10), where 0 is ‘no pain’ and 10 is ‘worst possible pain’, assessed at baseline, week 1, week 2, week 4 and week 12 [2].

• Disability: measured using the Roland Morris Disability Questionnaire 0–24 scale at baseline, week 1, week 2, week 4 and week 12 [2].

• Patient-generated measure of function: measured using the Patient-Specific Functional Scale (0 to 10 scale) at baseline, week 1, week 2, week 4 and week 12 [2].

• Global rating of change in back pain symptoms from onset: measured using the Global perceived change scale (−5 ‘vastly worse’ to 5 ‘completely recovered’) assessed at baseline, week 1, week 2, week 4 and week 12 [2].

• Sleep quality over the previous week: measured using a modification of item 6 of the Pittsburgh Sleep Quality Index (very bad, fairly bad, fairly good, very good) assessed at baseline, week 1, week 2, week 4 and week 12 [3].

• Quality of life: measured using the Short Form-12 version 2 (SF12v2) at baseline, week 4 and week 12 [4].

#### *Definition of process variables*

**Adherence:** Three measures of adherence will be considered. These are the number of tablets the participant reports they consumed per day until recovery or the end of the treatment period (28 days) as recorded in the daily medication diary; the number of tablets consumed by participants as assessed by counts of returned (remaining) tablets; and the proportion of tablets the participant reported they consumed of the recommended number of tablets.

The number of recommended tablets was 6 tablets per day in three doses until recovery or the end of the four week treatment period. Adherence was assessed at the week 4 follow-up point (28 days) and reported on a 0% to 100% numerical rating scale adapted from the Brief Adherence Rating Scale [5].

**Participant blinding to treatment:** At Week 12, participants were asked to answer the question ‘*Do you believe the medication in the red box was real paracetamol, the white was real paracetamol or that neither were real paracetamol*’. Their responses could be ‘red package contained real paracetamol; white package contained real paracetamol; or neither package contained real paracetamol.

**Satisfaction with treatment:** Participants were asked to rate their satisfaction with treatment at week 12 on a 0 to 6 likert scale, from 0 ‘not at all satisfied’ to 6 ‘extremely satisfied’.

**Concomitant treatments:** Participants were asked to record separately all medication and health-care services used for their back pain, other than that provided, for the study at week 4 and week 12. Information for each additional treatment was provided as free text often using variable terminology. These will be aggregated using a common terminology. Medications will be coded using the Anatomical Therapeutic Chemical Classification System at the fifth level. Other health services will be coded according to common provider types, for example specialist, hospital or emergency department presentation or admission, physiotherapy, chiropractic, massage therapy, other allied health, alternative medicine, and other.

Use of study rescue mediation, which could be provided by the participant’s general practitioner, were captured at weeks 1, 2 and 4 and will be presented separately.

**Safety:** The safety and tolerability of each paracetamol regimen will be considered. Information about serious adverse events (SAE) or adverse events (AE) were captured at weeks 1, 2, 4 and 12. SAEs, that is, death, life-threatening illness, hospitalization, substantial disability or incapacity, and congenital anomaly or birth defect, were investigated for possible association with the study medication at the time of reporting. An SAE committee determined and reported associations of SAEs with study medication as none, unlikely, possible, probable or definite. Unblinding and detailed investigation of the SAE was to be performed if an association with the study medication was deemed possible, probable or definite. As participants were asked to report all medical events or symptoms that occurred, irrelevant AEs are likely. For this reason, all AEs, along with SAEs, will be categorized using the World Health Organization International Classification of Diseases coding three digit codes (for example, A09) [6]. All AEs and SAEs will be described for each group in appendices.

### Analysis principles

Primary analyses will be conducted independently by two analysts who are blinded to group status. The results of the independent analyses will be compared and discrepancies in the results will be resolved. Analyses will be conducted using Stata and/or SAS. The analyses will be conducted using intention-to-treat (that is, analysed as randomized). All statistical tests will be two-tailed. Treatment effect for the primary outcome will be considered significant if *P* ≤0.05. For the secondary outcomes, treatment effect will be considered significant if *P* ≤0.01.

We will not impute values unless specified otherwise (see ‘Methods for handling missing data’). We will report the number of observations used in each analysis. Summaries of continuous variables that are normally distributed will be presented as means and SDs or medians and inter-quartiles for skewed data, whereas categorical variables will be presented as frequencies and percentages. This statistical analysis plan identifies pre-specified analyses only.

## Design issues

### General design

The PACE study is a three-arm randomized double blind double dummy placebo controlled trial. Consecutive patients or people from the community seeking care for acute low back pain were screened by primary care clinicians for eligibility.

### Treatment allocation

Eligible patients were randomized to one of three groups: time-contingent paracetamol dose regimen (plus placebo ‘as required’ paracetamol); ‘as required’ paracetamol (plus placebo time-contingent paracetamol); or a double placebo study arm (placebo time-contingent paracetamol plus placebo ‘as required’ paracetamol). When the study clinician determined a patient was eligible to enter the trial, the patient was supplied with a randomized treatment pack containing the study medicines. A research assistant then contacted the screened patient to confirm the patient’s eligibility and, after baseline assessment, instructed them to break the seal on the randomized treatment pack. Only at this point was the patient considered randomized to the trial.

### Sample size

Sample size calculations were based upon the median days to recovery being 14 in the time-contingent paracetamol group [7], assuming a median days to recovery of 17 days in comparison groups. A sample size of 550 individuals per arm (n = 1,650) has 80% power to detect a significant difference of at least three days in median time to recovery between each active group and the placebo group, allowing for up to 10% treatment non-compliance and a two-sided alpha of 0.05. As no prior knowledge is available regarding a meaningful effect of paracetamol on low back pain recovery, this effect size was based on consensus between investigators during the design of the study.

### Data collection and follow up

The different stages of data collection and follow-up are summarized in Table 1. Baseline assessment was conducted within 24 hours of referral from the practitioner and before commencing study treatment (day 1). Participants completed a daily pain diary until ‘sustained recovery’ or for 84 days (12 weeks), whichever came first. These data were used to determine the primary outcome (days to sustained recovery or days to first recovery). The primary outcome data and secondary outcome data were collected at week 1, week 2, week 4 and week 12. If a participant was not recovered by week four (day 28), additional collection points for primary data occurred every two weeks until recovery or week 12.

**Table 1:** Data collection and follow up stages

**Time point**	**Data captured**
Pre-randomisation	
Consultation with clinician	Inclusion/exclusion criteria; informed consent; type and date of consultation
Baseline assesment	Inclusion/exclusion criteria confirmed; date enrolled in the study; demographic data;
	Episode characteristics: duration of current symptoms, number of previous episode, presence of leg pain, days of reduced activity from back pain, feelings of depression, perceived risk of pain persistence, compensation status, work status, income and health insurance;
	Outcome variables: baseline pain intensity visual analogue scale, global perceived change scale (GPC), sleep quality (item 6 Pittsburg Sleep Quality Index), Patient Specific Functional Scale (PSFS), Roland Morris disability questionnaire (RMDQ), Credibility Expectancy Questionnaire, Short Form 12 (SF12v2)
Week 1	
Day 1 to 7	Daily pain rating scale and daily medication intake
Day 7	Adverse events, if participant returned to clinician and if offered rescue medication, pain intensity visual analogue scale, GPC, sleep quality, PSFS, RMDQ
Week 2	
Day 8 to 14	Daily pain rating scale and daily medication intake if not recovered prior
Day 14	Adverse events, if participant returned to clinician and if offered rescue medication, pain intensity visual analogue scale, GPC, sleep quality, PSFS, RMDQ
Week 4	
Day 15 to 28	Daily pain rating scale and daily medication intake if not recovered prior
Day 28	Adverse events, pain intensity visual analogue scale, GPC, sleep quality, PSFS, RMDQ, SF12v2, Hours absent from work per week (weeks 1–4), additional treatments used (weeks 1–4), Brief Adherence rating Scale.
Week 12	
Day 29 to 84	Daily pain rating scale until recovery or day 84 if not recovered prior
Day 84	Adverse events, pain intensity visual analogue scale, GPC, sleep quality, PSFS, RMDQ, SF12v2, hours absent from work per week (weeks 5–12), additional treatments used (weeks 5–12), satisfaction with treatment, treatment blinding questionnaire.

### Interim analysis

In the PACE study, paracetamol was used under its approved label use, therefore no interim analysis was conducted.

## Statistical analysis

### Trial profile

**Figure 1:**
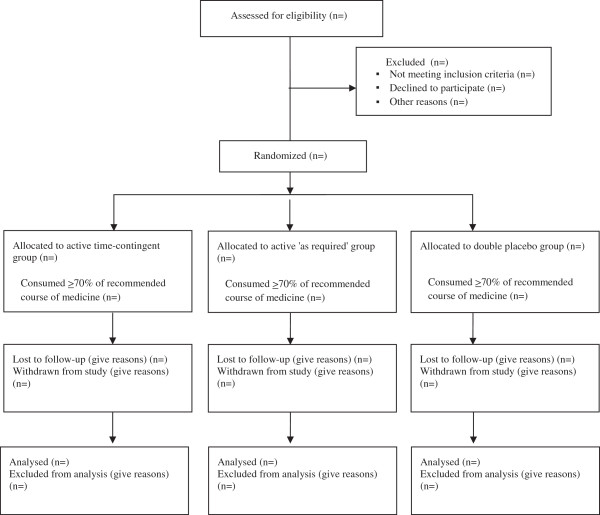
Consort flow diagram.

Flow of patients through the study will be displayed in a Consolidated Standards Of Reporting Trials (CONSORT) diagram. We will report the number of screened patients who met study inclusion criteria, reasons for exclusion of non-included patients, the number of participants randomized per group, and the number who completed follow-up, as shown in Figure 1.

### Data integrity

Basic checks will be conducted on all variables to identify data entry errors (for example by screening for empty cells and out-of-range values). Errors will be explored and corrected.

Data required to assess the primary outcome have been entered into two different study databases throughout the study. The following process will be conducted to ensure integrity of primary data:

1. The source data will be confirmed for every participant. The order of preference for which source of data will be used in the primary analysis is:

a. Pain scores recorded by the participant in returned pain diaries

b. Pain scores recorded directly online by the participant

c. Pain scores reported by the participant via telephone and transcribed by a research assistant into a paper copy of the participant’s diary or directly into online database

d. If all the above are missing, a single recovery date (or information about non-recovery) captured via correspondence with a participant. When only a single recovery date was attained by participants, research assistants confirmed that the participants ‘recovery’ had occurred for seven consecutive days of pain score of 0 or 1 out of ten.

e. If all the above were missing, a single date of recovery (or information about non-recovery of participants) was captured during correspondence with the participant’s treating clinician or nominated secondary contact person.

2. A random 10% sample of daily pain scores will be cross-checked between the pain diaries and data entered into the study database. If the upper bound of the 95% confidence interval of the error rate is greater than 10%, cross-checking of all participant’s diaries with entries made into study database (other than those that were recorded directly by the participants themselves to the online database) will take place. The error rate is the number of days for which there is an error over the total number of days data were recorded for each participant.

3. The primary outcome, number of days until recovery from low back pain, will be derived using two methods and compared via a statistical program using available daily pain data entered into the study or online database and manually by a research assistant using pain diary data and other sources (see ‘Data integrity: point 1) recorded in outcome assessment booklets. Discrepancies will be investigated and resolved before the final analysis.

An audit of secondary data will be conducted by cross-checking data written into participant outcome assessment booklets with data entered into the online database. A random 10% sample of participant booklets will be cross-checked. Any errors identified in this process will be corrected. If more than 10% of the data are in error, another 10% sample will be drawn for further checks. As an observation can only be checked once, the acceptable error rate for this new sample will reduce to 9%. If the error rate is again higher than the acceptable rate (that is, 9%), another 10% sample will be drawn for checking and the next acceptable error rate will reduce by 1% (that is, 8%). This process will continue until the observed error rate is below the acceptable threshold.

### Methods for handling missing data

The number of missing observations will be reported. Completeness of primary survival data will be calculated using the completeness index [8]. Should this exceed 10%, patterns of missing data will be investigated. Participants with no available outcome data will be censored at day one. Participants who are lost to follow-up will be censored on the day they last provided data. All other available primary data will be used until recovery or censoring. If any daily pain score is missing before the outcome ‘recovery for seven consecutive days’ has been established, we will assume that a 0 or 1 score for pain did not occur on the missing day/s, that is to say, no recovery. This is consistent with our line of questioning during data collection, where every effort was made to confirm that recovery (0 or 1 score for pain) had not occurred during periods of missing data or un-established recovery.

### Evaluation of demographics and baseline characteristics

The description of baseline characteristics listed below will be presented by treatment group. Categorical variables will be summarized by frequencies or denominators and percentages. Percentages will be calculated using the number of patients for whom data is available as the denominator. Denominators will be systematically reported (for example, nn/NN,%). Continuous variables will be summarized using standard measures of central tendency and dispersion, either mean and SD or median and interquartile range.

Demographics and baseline characteristics:

• Age at randomisation

• Gender

• Days since onset of pain

• Previous history of back pain (number of previous episodes)

• Presence of pain extending beyond the knee

• Number of days that the episode of pain caused reduced activity

• Depression sub-scale of The Örebro Musculoskeletal Pain Questionnaire

• Perceived risk of persisting pain from this episode

• Compensation status

• Other medications used

• Socioeconomic indicators (work, income, health insurance)

• Pain intensity (numerical rating scale)

• Global rating of change in back complaint since onset of back complaint

• Sleep quality (Item 6 of the Pittsburgh Sleep Quality Index)

• Disability (Roland Morris Disability Questionnaire)

• Function (Patient Specific Functional Scale)

• Credibility/Expectancy of treatment (Credibility Expectancy Questionnaire)

• Quality of Life (SF12v2)

### Process measures and concomitant treatments

Categorical and continuous measures listed below will be summarized. When indicated, data will be summarized per group. Again continuous variables will be summarized by use of standard measures of central tendency and dispersion, either mean and SD or median and interquartile range. Categorical variables will be summarized by frequencies or denominators and percentages.

Process variables and concomitant treatments:

• Estimated number of patients invited to participate

• Estimated number of patients eligible to participate

• Numbers and proportion of all eligible patients who chose to participate

• Numbers and proportion of all eligible patients who did not participate and (where available) reasons for not participating

• Number and proportion of participants who completed study follow-up, were lost to follow-up, withdrew from the study and reason for withdrawal

• Completeness of outcome survival data

• Adherence measures:

○ The number of tablets the participant reports they consumed per day until recovery or the end of the treatment period (28 days) as recorded in the daily medication diary;

○ The number of tablets consumed by participants as assessed by counts of returned (remaining) tablets;

○ The proportion of tablets the participant reported they consumed of the recommended number of tablets report at the end of week 4

• The proportion of participants having concomitant treatments per group

• Type and frequency of concomitant treatments

• The proportion of participants provided rescue medication.

### Primary analyses

A cox proportional hazards model will be used to assess the effect of treatment on outcome. The primary outcome is time from randomization to recovery. Censorship will occur on the day of the last follow-up, or when the patient was identified as recovered, whichever occurs earlier. Treatment will be a dummy coded variable representing treatment exposure (0 = placebo, 1 = as required and 2 = time-contingent). Adjustment will be made for baseline pain scores as this has consistently been identified as an important covariate [9].

Three primary comparisons will be conducted. To account for multiplicity we will employ the following closed test procedure recommended by Proschan and Waclawiw [10] to maintain an overall type 1 error rate of 0.05. First, a global Wald test will be used to assess the overall null hypothesis that all treatments are equally effective. If the global Wald test suggests that the effect of the treatment on at least one group is significantly different from the others, indicated by a *P* <0.05, the following pairwise comparisons will be conducted: time-contingent paracetamol will be compared to placebo; as required paracetamol will be compared to placebo; and time-contingent paracetamol will be compared to ‘as required’ paracetamol.

For these pairwise comparisons, again using the Wald test statistic, a *P-*value of 0.05 would indicate a significant treatment effect. Hazard ratios will be presented with their 95% confidence intervals along with median survival times (recovery) for each group with 95% confidence intervals.

The proportional hazards assumption will be tested with two checks. Firstly by observing survival curves; crossing of survival curves would indicate violation. Secondly, a time-dependent covariate will be introduced into the cox regression model. Beta values and significant interactions with In(time) will be inspected. If there is clear evidence of violation of the proportional hazards assumption, we will consider the effect of treatment on outcome using an unadjusted log rank test.

#### Secondary analyses

To test the effects of intervention on the secondary continuous outcomes (see ‘Definition of secondary outcomes’), between-group comparisons will be conducted using longitudinal mixed models combining all available post-randomization measurements. A log-binomial regression will be used for categorical outcomes (sleep quality) with robust Poisson regression as backup in case of convergence issues [11]. The main test will compare the weighted average combining estimates from the four follow-ups between the treatment arms. If this is statistically significant we will proceed to test pairwise differences between groups for each follow-up separately.

The independent variables will include a dummy-coded variable indicating group membership, the time at which the measurement was taken (week 1, week 2, week 4 or week 12 post randomization), and the four time-by-group interactions. Adjustment will be made for baseline outcomes scores. The effect of paracetamol at each of the four follow-up time points is estimated with the relevant interaction term. The model will incorporate random intercepts to account for the dependence of repeated measures.

### Sensitivity analyses

#### *Recovery definitions*

We will explore the influence of different recovery definitions on the primary outcome of the study. A second definition, the first day a participant reports a 0 or 1 out of 10 on a 0 to 10 numerical rating scale, was previously reported by Hancock *et al*. [7] to result in a similar event rate of recovery.

#### *Adjusting for prognostic variables*

In addition to baseline pain, the effect of baseline characteristics, which have been suggested to influence recovery [9,12], will be assessed. For primary outcomes, the Cox regression model previously described will be extended to include the number of previous episodes of back pain and the duration of the current episode. Hazard ratios and 95% confidence intervals will be calculated for treatment effect and each covariate. The proportional hazards assumption will be tested as previously described. Stratification will be considered in the event of violation of the proportional hazards assumption. The covariates ‘the number of previous episodes of back pain’ and ‘the duration of the current episode’ will be added to the models outlined for secondary analyses i to assess their influence on secondary outcomes.

#### *Imputation of missing data*

The influence of different imputation methods for missing data will be assessed. Imputation of best case scenarios (for example, missing data point equals a recovered value (0 or 1)) will be compared to the proposed method (worst case; missing value equals a non-recovered value (>1)) in ‘Methods for handling missing data’. These analyses will be conducted on observations where daily scores are available, but some days are missing. They will not be conducted where only a single date of recovery has been recorded and daily pain scores are available (see ‘Data integrity’).

### Evaluation of serious adverse events and adverse events

The Fisher exact test will be used to compare the incidence of any AEs (as coded by the World Health Organization International Classification of Diseases) between groups. This test will be used as the event rate of AEs is expected to be low.

SAEs, when reported, were systematically investigated for potential association with the study treatment. No formal analysis will be conducted to compare SAEs between groups as the event rate is extremely low. All SAEs will be reported per group.

### Table and figure shells

Additional file 1: Table S1 will report all collected baseline characteristics of participants by treatment group. Figure 1 will display the consort flow diagram. Figure two (not provided) will display Kaplan-Meier survival curves for each treatment group for recovery to 84 days (12 weeks), with separate panels for each definition of recovery. This figure will also detail median recovery time per group with 95% confidence intervals and *P-*values for primary comparisons between groups. Additional file 1: Table S2 will report secondary outcome variables by treatment group and effects of treatment. Additional file 1: Table S3 will report process measures and concomitant treatments by treatment group and were appropriate results of statistical comparisons between groups. All adverse events will be described by treatment group in the manuscript appendices. Results from sensitivity analyses will be reported in the manuscript. New information revealed by sensitivity analyses will displayed graphically or in tabulation in the manuscript. Otherwise results of the sensitivity analyses will be tabulated in appendices.

## Abbreviations

AE: Adverse events; SAE: Serious adverse events; SD: Standard deviation; SF12v: Short Form 12 version 2.

## Competing interests

The authors declare no other competing interests.

## Authors’ contributions

CW drafted the manuscript and participated in the design of the study, and coordination and acquisition of data. Authors CM, JL, AM, RD and MH conceived the study and secured study funding, and participated in the design of the study and coordination and acquisition of data. LB participated in the design of the study and is coordinating statistical analyses. CL participated in the design of the study, and coordination and acquisition of data. All authors were involved in the analysis planning and helped draft the manuscript. All authors read and approved the final manuscript.

## Authors’ information

CW, PhD, honorary research fellow, CM, PhD, Director and Professor of Physiotherapy, JL, PhD, Principal Research Fellow, and CL, PhD, Senior Research Fellow are from the Musculoskeletal Division of the George Institute for Global Health and Sydney Medical School, The University of Sydney, Sydney, Australia. AM, PhD, is Professor of Pharmacy, The University of Sydney, Sydney, Australia. MH, PhD, is Senior Lecturer, Faculty of Human Sciences, Macquarie University, Sydney, Australia. RO, PhD, is Professor, Department of Clinical Pharmacology & Toxicology, University of New South Wales, Sydney, Australia. LB is Director, Statistics and Data Management Division, the George Institute for Global Health, The University of Sydney, Sydney, Australia.

## Supplementary Material

Additional file 1: Table S1 Baseline characteristics. **Table S2**: Secondary low back pain outcomes. **Table S3**: Effects of treatment, secondary outcomes. **Table S4**: Process measures. Click here for file
