# Supplementary material for: PACE – the first placebo controlled trial of paracetamol for acute low back pain: statistical analysis plan
Source: Trials. 2013 Aug 9;14:248. doi: 10.1186/1745-6215-14-248 (PMC3750911; doi:10.1186/1745-6215-14-248)
Supplement: Additional file 1: Table S1 — Baseline characteristics. Table S2: Secondary low back pain outcomes. Table S3: Effects of treatment, secondary outcomes. Table S4: Process measures. [file 1745-6215-14-248-S1.docx]

Table 1: Baseline characteristics

| **Participant characteristics** | Time-contingent | As required | Placebo |
| --- | --- | --- | --- |
| Age years | mean (SD) | mean (SD) | mean (SD) |
| Male gender | n/N (%) | n/N (%) | n/N (%) |
| Health insurance | n/N (%) | n/N (%) | n/N (%) |
| Income per annum (household) |  |  |  |
| Under $33,800 | n/N (%) | n/N (%) | n/N (%) |
| $33,800 – 88,399 | n/N (%) | n/N (%) | n/N (%) |
| $88,400 – 207,999 | n/N (%) | n/N (%) | n/N (%) |
| >$208,000 | n/N (%) | n/N (%) | n/N (%) |
| Employment | n/N (%) | n/N (%) | n/N (%) |
| Use of medication for other conditions | n/N (%) | n/N (%) | n/N (%) |
| **Episode characteristics** |  |  |  |
| Duration of symptoms days | mean (SD) | mean (SD) | mean (SD) |
| Number of previous episodes | median (IQR) | Median (IQR) | median (IQR) |
| Leg pain beyond the knee | n/N (%) | n/N (%) | n/N (%) |
| Number of days reduced activity | mean (SD) | mean (SD) | mean (SD) |
| Feelings of depression in the last week | mean (SD) | mean (SD) | mean (SD) |
| Perceived risk of persistent pain | mean (SD) | mean (SD) | mean (SD) |
| Current back pain episode compensable | n/N (%) | n/N (%) | n/N (%) |
| Current pain intensity | mean (SD) | mean (SD) | mean (SD) |
| Disability, RMDQ score | mean (SD) | mean (SD) | mean (SD) |
| Global perceived improvement, | mean (SD) | mean (SD) | mean (SD) |
| Poor sleep quality in last week | n/N (%) | n/N (%) | n/N (%) |
| Quality of Life, Physical Component Score | mean (SD) | mean (SD) | mean (SD) |
| Quality of Life, Mental Component Score | mean (SD) | mean (SD) | mean (SD) |
| Treatment credibility score* | mean (SD) | mean (SD) | mean (SD) |
| Treatment expectation score* | mean (SD) | mean (SD) | mean (SD) |

* P <0.01

Table 2: Secondary low back pain outcomes

|  | Unadjusted outcome measures | | |
| --- | --- | --- | --- |
|  | Time-contingent | 'As required' | Placebo |
| Pain |  |  |  |
| Week 1 | mean (SD) | mean (SD) | mean (SD) |
| Week 2 | mean (SD) | mean (SD) | mean (SD) |
| Week 4 | mean (SD) | mean (SD) | mean (SD) |
| Week 12 | mean (SD) | mean (SD) | mean (SD) |
| Disability (RMDQ) |  |  |  |
| Week 1 | mean (SD) | mean (SD) | mean (SD) |
| Week 2 | mean (SD) | mean (SD) | mean (SD) |
| Week 4 | mean (SD) | mean (SD) | mean (SD) |
| Week 12 | mean (SD) | mean (SD) | mean (SD) |
| Global perceived change |  |  |  |
| Week 1 | mean (SD) | mean (SD) | mean (SD) |
| Week 2 | mean (SD) | mean (SD) | mean (SD) |
| Week 4 | mean (SD) | mean (SD) | mean (SD) |
| Week 12 | mean (SD) | mean (SD) | mean (SD) |
| Poor Sleep quality |  |  |  |
| Week 1 | n/N (%) | n/N (%) | n/N (%) |
| Week 2 | n/N (%) | n/N (%) | n/N (%) |
| Week 4 | n/N (%) | n/N (%) | n/N (%) |
| Week 12 | n/N (%) | n/N (%) | n/N (%) |
| SF12v2 (PCS) |  |  |  |
| Week 1 | mean (SD) | mean (SD) | mean (SD) |
| Week 2 | mean (SD) | mean (SD) | mean (SD) |
| Week 4 | mean (SD) | mean (SD) | mean (SD) |
| Week 12 | mean (SD) | mean (SD) | mean (SD) |
| SF12v2 (MCS) | mean (SD) | mean (SD) | mean (SD) |
| Week 1 | mean (SD) | mean (SD) | mean (SD) |
| Week 2 | mean (SD) | mean (SD) | mean (SD) |
| Week 4 | mean (SD) | mean (SD) | mean (SD) |
| Week 12 | mean (SD) | mean (SD) | mean (SD) |

Table 3: Effects of treatment, secondary outcomes

|  | Effects of treatment | | |
| --- | --- | --- | --- |
|  | Time-contingent vs placebo | As-required vs placebo | Time contingent vs as required |
| Pain |  |  |  |
| Week 1 | mean (99%CI) | mean (99%CI) | mean (99%CI) |
| Week 2 | mean (99%CI) | mean (99%CI) | mean (99%CI) |
| Week 4 | mean (99%CI) | mean (99%CI) | mean (99%CI) |
| Week 12 | mean (99%CI) | mean (99%CI) | mean (99%CI) |
| Overall | mean (99%CI) | mean (99%CI) | mean (99%CI) |
| Disability (RMDQ) |  |  |  |
| Week 1 | mean (99%CI) | mean (99%CI) | mean (99%CI) |
| Week 2 | mean (99%CI) | mean (99%CI) | mean (99%CI) |
| Week 4 | mean (99%CI) | mean (99%CI) | mean (99%CI) |
| Week 12 | mean (99%CI) | mean (99%CI) | mean (99%CI) |
| Overall | mean (99%CI) | mean (99%CI) | mean (99%CI) |
| Global perceived change |  |  |  |
| Week 1 | mean (99%CI) | mean (99%CI) | mean (99%CI) |
| Week 2 | mean (99%CI) | mean (99%CI) | mean (99%CI) |
| Week 4 | mean (99%CI) | mean (99%CI) | mean (99%CI) |
| Week 12 | mean (99%CI) | mean (99%CI) | mean (99%CI) |
| Overall | mean (99%CI) | mean (99%CI) | mean (99%CI) |
| Poor Sleep quality |  |  |  |
| Week 1 | risk ratio (95%CI) | risk ratio (95%CI) | risk ratio (95%CI) |
| Week 2 | risk ratio (95%CI) | risk ratio (95%CI) | risk ratio (95%CI) |
| Week 4 | risk ratio (95%CI) | risk ratio (95%CI) | risk ratio (95%CI) |
| Week 12 | risk ratio (95%CI) | risk ratio (95%CI) | risk ratio (95%CI) |
| SF12v2 (PCS) |  |  |  |
| Week 1 | mean (99%CI) | mean (99%CI) | mean (99%CI) |
| Week 2 | mean (99%CI) | mean (99%CI) | mean (99%CI) |
| Week 4 | mean (99%CI) | mean (99%CI) | mean (99%CI) |
| Week 12 | mean (99%CI) | mean (99%CI) | mean (99%CI) |
| Overall | mean (99%CI) | mean (99%CI) | mean (99%CI) |
| SF12v2 (MCS) | mean (99%CI) | mean (99%CI) | mean (99%CI) |
| Week 1 | mean (99%CI) | mean (99%CI) | mean (99%CI) |
| Week 2 | mean (99%CI) | mean (99%CI) | mean (99%CI) |
| Week 4 | mean (99%CI) | mean (99%CI) | mean (99%CI) |
| Week 12 | mean (99%CI) | mean (99%CI) | mean (99%CI) |
| Overall | mean (99%CI) | mean (99%CI) | mean (99%CI) |

Table 4: Process measures

| **Variable** | Time-contingent | As required | Placebo |
| --- | --- | --- | --- |
| Adherence – |  |  |  |
| tablets consumed each day, median (IQR) | x | x | x |
| tablets consumed over study, median (IQR) | x | x | x |
| consumed >70% of recommended dose, % yes (n) | x | x | x |
| Participants receiving rescue medications, % yes (n) | x | x | x |
| Participants using other medications, % yes (n) | x | x | x |
| Participants using other services, % yes (n) | x | x | x |
| Participants reporting an SAE, % yes (n) | x | x | x |
| Participants reporting an AE, % yes (n) | x | x | x |
| Assessment of blinding, % correct (n) | x | x | x |
| Satisfaction with treatment | x | x | x |

**P <0.05

Adherance measures: (i) The number of tablets the participant reports they consumed per day until recovery or the end of the treatment period (28 days) as recorded in the daily medication diary; (ii) The number of tablets consumed by participants as assessed by counts of returned (remaining) tablets; (iii) The proportion of tablets the participant reported they consumed of the recommended number of tablets (the number of recommended tablets was 6 tablets per day in three doses until recovery or the end of the four week treatment period). This is assessed at the week four follow-up point (28 days) and reported on a 0-100 (%) visual analogue scale; adapted from the Brief Adherence Rating Scale.
